# LAMPOX: A Portable and Rapid Molecular Diagnostic Assay for the Epidemic Clade IIb Mpox Virus Detection

**DOI:** 10.3390/diagnostics15151959

**Published:** 2025-08-04

**Authors:** Anna Rosa Garbuglia, Mallory Draye, Silvia Pauciullo, Daniele Lapa, Eliana Specchiarello, Florence Nazé, Pascal Mertens

**Affiliations:** 1 Virology Laboratory, National Institute for Infectious Diseases “Lazzaro Spallanzani” IRCCS, 00149 Rome, Italy; annarosa.garbuglia@inmi.it (A.R.G.); daniele.lapa@inmi.it (D.L.); eliana.specchiarello@inmi.it (E.S.); 2 Coris BioConcept, Crealys Science Park, 5032 Gembloux, Belgium; mallory.draye@corisbio.com (M.D.); florence.naze@corisbio.com (F.N.); pascal.mertens@corisbio.com (P.M.)

**Keywords:** Mpox, point of care (POC), molecular diagnostics, LAMP (loop-mediated isothermal amplification), comparative analysis

## Abstract

The global spread of Mpox virus (MPXV) underscores the urgent need for rapid, field-deployable diagnostic tools, especially in low-resource settings. We evaluated a loop-mediated isothermal amplification (LAMP) assay, termed LAMPOX, developed by Coris BioConcept. The assay was tested in three formats—two liquid versions and a dried, ready-to-use version—targeting only the ORF F3L (Liquid V1) or both the ORF F3L and N4R (Liquid V2 and dried) genomic regions. Analytical sensitivity and specificity were assessed using 60 clinical samples from confirmed MPXV-positive patients. Sensitivity on clinical samples was 81.7% for Liquid V1 and 88.3% for Liquid V2. The dried LAMPOX assay demonstrated a sensitivity of 88.3% and a specificity of 100% in a panel of 112 negative controls, with most positive samples detected in under 7 min. Additionally, a simplified sample lysis protocol was developed to facilitate point-of-care use. While this method showed slightly reduced sensitivity compared to standard DNA extraction, it proved effective for samples with higher viral loads. The dried format offers key advantages, including ambient-temperature stability and minimal equipment needs, making it suitable for point-of-care testing. These findings support LAMPOX as a promising tool for rapid MPXV detection during outbreaks, especially in resource-limited settings where traditional PCR is impractical.

## 1. Introduction

Mpox virus (MPXV), formerly known as Monkeypox virus, is a double-stranded DNA (dsDNA) virus with a genome of approximately 197 kbp [1]. It belongs to the *Poxviridae* family, *Chordopoxvirinae* subfamily, and the *Orthopoxvirus* (OPXV) genus, which also includes Variola virus (VARV) (*Orthopoxvirus variola*), Cowpox virus (CPXV) (*Orthopoxvirus cowpox*), Vaccinia virus (VACV) (*Orthopoxvirus vaccinia*), Camelpox virus (CMLV) (*Orthopoxvirus camelpox*), Taterapox virus (TATV) (*Orthopoxvirus taterapox*), and Ectromelia virus (ECTV) (*Orthopoxvirus ectromelia*). Members of this genus exhibit immunological cross-reactivity [2].

Mpox was first identified in 1958 in a laboratory monkey at a research facility in Copenhagen, Denmark. However, the first human cases of Mpox were reported in the Democratic Republic of the Congo (DRC) in 1970 [3]. It is considered a zoonotic disease, having been isolated in non-human primates, African squirrels, and various rodents; moreover, person-to-person transmission also contributes to the spread of the disease [4,5,6]. Mpox is endemic in some regions of West and Central Africa, including Nigeria, Liberia, Sierra Leone, Gabon, Ivory Coast, the Central African Republic, DRC, Benin, and Cameroon [7]. Until 1980, only sporadic cases of Mpox were reported, as smallpox vaccination provided cross-protection against Mpox, limiting its transmission among humans. Following the cessation of smallpox vaccination programs, several Mpox outbreaks have occurred in endemic regions. For example, recurrent outbreaks occur in the DRC, accounting for approximately 98% of all Mpox cases up to 2022 [8].

The first Mpox outbreak in a non-endemic region occurred in 2003 in the USA [9]. Between 2018 and 2021, other sporadic travel-related cases were reported in the United Kingdom, Singapore, Israel, and the USA [10]. In May 2022, the biggest Mpox outbreak began, characterized by international spread. In July 2022, the World Health Organization (WHO) declared Mpox a Public Health Emergency of International Concern [11]. The first cases were reported in the United Kingdom on 7 May 2022. Subsequently, several other countries, including Italy, France, and the Netherlands, also detected Mpox cases [12,13,14].

One year later, a new epidemic arises in the Democratic Republic of the Congo, involving both clade Ia and clade Ib strains. In 2024, more than 13,000 cases and 43 deaths were reported, with an additional 2000 cases recorded in the first months of 2025 [15].

As of February 2025, the WHO continues to classify Mpox as a global health emergency [16], resulting in approximately 140,000 reported cases across 132 countries, with 317 deaths [17].

The main symptoms of Mpox are generic, common to other illnesses such as the flu or malaria. These include fever, myalgia, headache, swollen lymph nodes, and fatigue [18]. A characteristic rash typically appears after the onset of febrile symptoms [19]. The incubation period ranges from 4 to 21 days, with an average of 6–13 days [13,20]. Infected individuals may become contagious 1–4 days before symptom onset [6].

Mpox infection usually resolves within 2–4 weeks. However, prolonged viral persistence has been observed in pregnant women, children, and immunocompromised individuals. Since vaccination is still limited to healthcare workers, rapid and early detection is currently the most effective strategy to limit transmission.

Transmission occurs through direct contact with skin lesions or via the oral-respiratory route, as the airway epithelium is a primary target for initial viral replication [21]. Following primary infection, the virus may disseminate to the tonsils, spleen, liver, kidneys, intestines, and skin, with keratinocytes, fibroblasts, and endothelial cells serving as permissive hosts [21]. In men who have sex with men (MSM), genital and anal lesions are frequently observed and may facilitate sexual transmission of Mpox. Additional potential transmission routes include respiratory droplets, body fluids, contaminated fomites, and viral shedding through feces and semen [22,23]. Notably, clade Ib, associated with a recent outbreak in DRC since September 2023, has shown higher transmissibility and disease burden compared to other clades. Genomic analyses revealed mutations, including an OPG032 gene deletion and APOBEC3-driven mutations, which may contribute to increased viral replication and sustained human-to-human transmission [24].

One of the key strategies to controlling the spread of Mpox is the rapid detection of positive cases combined with efficient contact tracing. As Mpox-infected individuals can be contagious before the onset of clinical symptoms, highly sensitive diagnostic assays are essential to minimize false-negative results. Molecular assays represent the main tool for early detection of Mpox infection, and due to their high sensitivity, they are recommended by the WHO [25].

A variety of clinical samples can be used for Mpox detection, including swabs from skin lesions, oral and pharyngeal mucosa, the anus, the genitals, or the conjunctiva. Blood samples are particularly useful during the early phase of infection, as viremia may be present even before specific symptoms appear [26].

Several real-time PCR assays with varying levels of specificity and sensitivity are currently available [27,28,29,30]. However, these methods typically require specialized laboratory equipment and a cold chain for reagent preservation.

An alternative and promising technique with comparable diagnostic performance is loop-mediated isothermal amplification (LAMP). This method utilizes auto-cycling strand displacement DNA synthesis by *Bst*DNA polymerase to amplify DNA fragments without the need for a thermocycler device. Due to its operational simplicity and minimal equipment requirements, LAMP is particularly suitable for use in low-resource areas [31]. In this paper, we show the performance of LAMPOX assay (Coris BioConcept, Gembloux, Belgium), a dried reagent LAMP-based assay that does not require cold chain storage, on a panel of positive and negative samples collected in a European laboratory, Istituto Nazionale per le Malattie Infettive “Lazzaro Spallanzani” (INMI), Italy.

## 2. Materials and Methods

### 2.1. Specimen Collection

Nasopharyngeal swabs (TNF) (n = 9) and skin lesions (n = 51) were collected in universal transport media (UTM, RT-UTM Copan, Brescia, Italy) at INMI L. Spallanzani Hospital from patients suspected to be infected by MPXV during Mpox outbreak between May and December 2022. All samples were confirmed PCR-positive for Mpox (described in Section 2.3). Each specimen was divided into two aliquots: one half was subjected to nucleic acid extraction, while the remaining material was stored at −80 °C. Ethical approval was obtained from the local ethical committee (ethical approval code 40z/2022). In addition, anal swabs (n = 60) and oral rinse (ROC, n = 52) from HPV DNA-positive and Mpox-negative individuals were included as negative controls to assess the specificity of the CORIS LAMP assay.

### 2.2. Propagation and Thermal Inactivation of MPXV Viral Stocks

MPXV viral stocks were propagated in Vero E6 cells (ATCC^®^ CRL-1586™, Manassas, VA, USA) using the isolate hMpxV/Italy/un-INMI-Pt2/2022 (clade IIb, lineage B.1). Viral titers were determined to be 6.81 Log TCID_50_/mL. To assess viral infectivity, 15 serial 0.5-Log dilutions of the viral supernatant (starting from undiluted) were prepared in MEM supplemented with 2% fetal bovine serum (FBS) and inoculated onto sub-confluent Vero E6 cell monolayers seeded in 96-well plates. Cultures were incubated at 37 °C in a 5% CO_2_ humidified atmosphere, and cytopathic effect (CPE) was evaluated microscopically at 5 days post infection. The TCID_50_/mL (50% Tissue Culture Infective Dose) was calculated using the Reed–Muench method. To confirm productive infection and viral replication, two sequential passages were performed in sub-confluent Vero E6 cells. The cells were washed twice and inoculated with virus diluted 1:20 (50 µL in 1 mL of serum-free MEM). Following adsorption for 1 h at 37 °C with 5% CO_2_, 4 mL of MEM supplemented with 2% FBS was added to each culture without removing the inoculum. The cultures were observed daily for the appearance of CPE for up to 5 days post infection. For heat inactivation, aliquots of MPXV stock were incubated at 70 °C for 5 min using a thermoblock machine, followed by immediate cooling on ice for 10 min. Mock-inactivated controls (MEM only) underwent identical thermal treatment and were included in all inactivation assays. Each inactivation condition was performed at least in duplicate. To verify complete inactivation, treated viral preparations were subjected to 15 serial 0.5 Log dilution and inoculated onto sub-confluent Vero E6 cells in 96-well plates under the same conditions as for infectivity assays. Cells were monitored for CPE for up to 5 days post infection to detect any residual infectious particles.

### 2.3. Mpox PCR Reference Assay

The DNA from all types of samples was extracted from 400 µL of specimen using QIASYMPHONY automated instruments, and they were eluted in 60 µL elution buffer provided by QIAGEN (QIAGEN, Hilden, Germany). The extracted DNA from the viral stock was then quantified using a spectrophotometer. Serial dilutions of quantified DNA were used to calculate the analytical sensitivity of the assay.

The positivity for Mpox was previously evaluated with a homemade PCR. The reference test that was used in this study was based on the reaction developed by Li et al. [32] for general detection of Mpox. The primers and probe used were the following: forward primer (5′-GGAAAATGTAAAGACAACGAATACAG); reverse primer (5′-GCTATCACATAATCTGGAAGCGTA); probe sequence (5′FAM-AAGCCGTAATCTATGTTGTCTATCGTGTCC-3′BHQ1). Primers and probe were incorporated into a single assay together with primers and probes targeting human RNase P gene (fluorophore TX615) as an internal control (IC). PCR was performed on Rotorgene with the following conditions: one cycle at 95 °C for 3 min; 45 cycles at 95 °C for 15 s, at 59 °C for 45 s, and at 72 °C for 15 s. To monitor potential cross-contamination during the PCR step, a negative control (PCR-grade water) was added to a tube containing the reaction mixture.

The internal control was included in each reaction to monitor sample integrity and test validity. The samples were considered positive when the Mpox gene was detected and negative when the Mpox gene target was not amplificated and the IC was detected. The positivity was calculated considering the Ct values: a Ct value <40 was considered as a positive result.

### 2.4. LAMP Assay

Three mix formulations of the LAMP assay, two different liquid versions (frozen reagents) and a dried version, were employed for the detection of MPXV, all based on fluorometric detection using a DNA intercalating dye. In all versions, primers were designed to target the MPXV genome: the first liquid version targets the ORF F3L region, while both the second liquid version and the dried version include primers targeting the ORF F3L and N4R regions. For the liquid formulations, the reaction mix was freshly prepared by combining a LAMP mastermix (containing enzymes, buffer, dNTPs, and the intercalating dye) with the primer mix. Additionally, the second liquid version included 1 μL of a LAMP additive. Specifically, the reaction mix for the first liquid formulation consisted of 10 μL of mastermix and 5 μL of primer mix, while the second liquid version used 10 μL of mastermix, 4 μL of primer mix, and 1 μL of additive. For the dried version, the reaction components were pre-aliquoted and lyophilized into 0.2 mL PCR strip tubes. To reconstitute the dry mix, 15 μL of rehydration solution was added directly to the pellet in each tube. For all three formulations, 5 μL of extracted DNA or MPXV positive control was added to the 15 μL of reaction mix, reaching a final reaction volume of 20 μL. A no template control (NTC) was prepared by adding 5 μL of nuclease-free water. Amplification was carried out using a real-time thermocycler (Rotorgene, QIAGEN) under the following cycling conditions: 60 cycles at 63 °C for 30 s corresponding to a total time reaction of 30 min. The fluorescence signal was collected at each of the 60 repeats. After the amplification step, a melting curve analysis was performed by applying a temperature gradient from 63 °C to 95 °C at a rate of 0.5 °C every 5 s, and fluorescence data were collected at each temperature increment. The upper and lower limits of detection of the tests were 10^4^ and 153.6 copies/μL, respectively. No hook effect could be demonstrated in this range of concentrations. However, the possibility of a hook effect occurring outside the tested concentrations could not be excluded.

### 2.5. Sample Lysis Protocol

A chemical lysis protocol using a proprietary buffer was employed by Coris BioConcept (Gembloux, Belgium) to release viral nucleic acids from clinical or transport medium-diluted samples without requiring a subsequent RNA or DNA purification step. Due to the experimental nature and non-commercial status of this protocol, detailed composition is not available at this stage. The method was evaluated on 21 Mpox-positive samples selected to represent a wide range of Ct values obtained by the homemade PCR, allowing a preliminary assessment of its performance across varying viral loads. For each reaction, 30 μL of the prepared viral lysis buffer was combined with 10 μL of sample in a sterile, nuclease-free 1.5 mL microcentrifuge tube. The mixture was vortexed briefly and collected at the bottom of the tube by a quick spin. Samples were then incubated at 95 °C for 5 min in a pre-heated thermoblock. Following heat treatment, tubes were centrifuged at 3000× *g* for 30 s at room temperature to facilitate complete collection of the lysate. The resulting lysate was used directly for LAMP-based nucleic acid amplification assays.

### 2.6. Statistical Analysis

Comparison between the results of each assay was performed using the GRAPHPAD Prism 9 software (www.graphpad.com, accessed on 20 June 2025). The sensitivity (95% confidence interval) was calculated based on the number of true positive and false negative samples by using a suitable program. Confidence intervals for sensitivity are “exact” Clopper–Pearson confidence intervals.

## 3. Results

### 3.1. LAMP Assay Design for Mpox Virus Detection

LAMP assays were designed to target clades I and II (West African and Congo Basin clades). Sets of in-house primers (F3, B3, FIP, BIP, and Loops) were designed based on the analysis of sequences available from the National Centre for Biotechnology Information (NCBI) database. The ORF F3L region was targeted first and then the N4R region to increase the sensitivity of the LAMP assay. Two assays in liquid version were developed, with the first targeting only the ORF F3L region (Liquid V1) and the second targeting the ORF F3L and N4R regions (Liquid V2).

LAMP assay conditions (Mg^2+^, dNTP, primer, and additive concentrations) were optimized first with a synthetic DNA corresponding to the target part of the ORF F3L and then with genomic DNA extracted from an inactivated hMpxV/Italy/un-INMI-Pt2/2022 MPXV culture.

### 3.2. Performances of the Two Liquid Versions of the MPXV LAMP Assays for Clinical Samples

The sensitivity of the two versions of the liquid MPXV LAMP assays was assessed with DNA extracted from the collection of nasopharyngeal swabs and skin lesion specimens (total of n = 60). These samples were selected to correspond to PCR Ct values ranging from 12.85 to 35.91.

**Figure 1:**
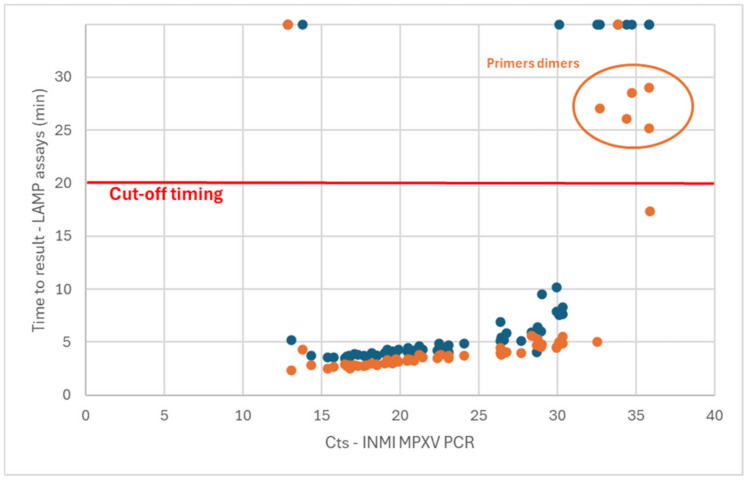
Comparison of the two versions of the liquid MPXV LAMP assay. Version V1 (ORF F3L) in blue dots and version V2 (ORF F3L and N4R) in orange dots. A time-to-result above 30 min (maximal duration of the assay) means that no amplification was observed for this sample.

Using the Liquid V1 assay (ORF F3L only), 49/60 samples were detected as positive [81.7% (70.1 to 89.4%, 95% confidence interval)] (Figure 1—blue dots). From the 11 false-negative results, 9 were samples harboring a Ct value in PCR of above 30 (all skin lesions). Surprisingly, the two other false-negative detections were samples with very low Ct values in PCR (12.85 and 13.80, all skin lesions). A retest was carried out for these two samples, with the same observation. This could have been caused by a hook effect, where highly concentrated samples could not be detected as expected.

Using the Liquid V2 assay (2 gene targets), 58/60 samples were detected in less than 30 min (Figure 1—orange dots). Among these detected samples, it was observed that the melting temperatures of amplicons associated with the samples with a time-to-result above 25 min did not correspond with that of the other positive samples. However, this profile of melting curves was sometimes observed in no template control (NTC). This observation could be due to primer dimers forming when low amounts of matrix are present in the sample. These five samples were thus associated with a false-positive detection, leading to the setting of a cutoff value of 20 min for the determination of the positivity. Based on this cutoff value, 53/60 samples were detected as positive [88.3% (77.8 to 94.2%, 95% confidence interval)]. One of the samples with a low Ct value (12.85) that was not detected with the Liquid V1 assay was also not detected with the Liquid V2 assay. It can also be observed that the time-to-result obtained with the Liquid V2 assay was shorter than that of the Liquid V1 version (average of 1.3 min for samples with Ct values in PCR ranging from 15 to 31).

The analytical sensitivity for the Liquid V1 and Liquid V2 LAMPOX assay was determined using dilutions of hMpxV/Italy/un-INMI-Pt2/2022 genome copies. The limit of detection of these assays was defined to 300 and 150 viral genome copies/reaction, respectively.

### 3.3. Performances of the Dry Version of the MPXV LAMP Assay

For field use, a stabilized version of the LAMP assay may be required. Based on the results obtained during the comparison of the two versions of the liquid LAMP assay, version V2 (ORF F3L and N4R regions) was selected for the next step involving the development of a dried version of the assay (dry LAMPOX assay).

The clinical sensitivity of the dry LAMPOX assay was assessed with the same DNA extracts previously analyzed with the liquid versions (from nasopharyngeal swabs and skin lesion specimens, total of n = 60).

**Figure 2:**
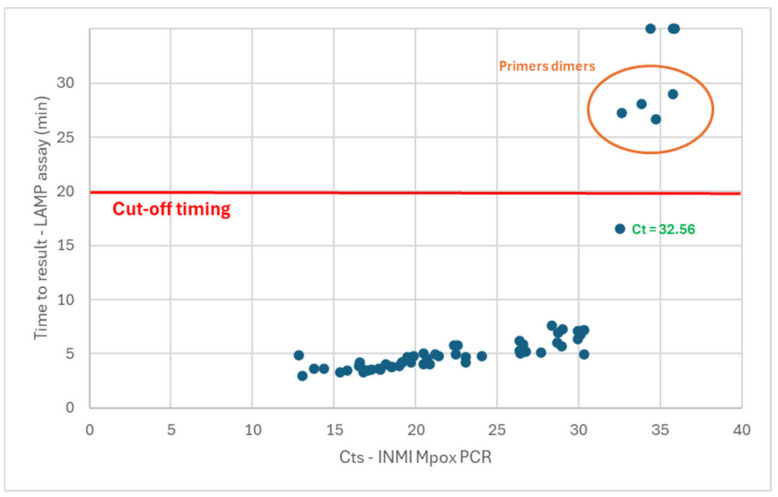
Sensitivity performances of the dry version of the LAMP assay. A time-to-result of above 30 min (maximum duration of the assay) means that no amplification was observed for this sample.

A total of 58/60 samples were detected, with a time-to-result of under 30 min (Figure 2). As previously observed for the Liquid V2 assay, the melting temperatures associated with the samples showing a time-to-result of above 25 min were not specific to the expected amplicons (with primer dimers forming when low amounts of matrix are present in the sample). These five samples were thus associated with a false-positive detection. These observations confirmed the need to set a cutoff value of 20 min for the determination of the positivity. Based on these observations, 53/60 samples were detected as positive for a sensitivity of 88.3% (77.8 to 94.2%, 95% confidence interval). In contrast with the liquid versions of the assay, the two samples with low Ct values in PCR (12.85 and 13.80) were detected as positive. With this version of the test, 100% of the samples with Ct values in PCR of up to 32.56 were detected in a time-to-result under the cutoff of 20 min (with 90.6% of these samples detected in less than 7 min).

The analytical sensitivity for the dry LAMPOX assay was determined using dilutions of hMpxV/Italy/un-INMI-Pt2/2022 genome copies. The limit of detection of the assay was defined as 175 viral genome copies/reaction.

### 3.4. Specificity of the Dry Version of the MPXV LAMP Assay

To assay the specificity of the dry LAMPOX assay, DNA extracts from samples characterized as MPXV negative and HPV DNA positive by PCR [anal swabs (n = 60) and oral rinse (ROC, n = 52)] were analyzed. None of the 112 tested DNA samples presented an amplification curve during the 30 min of the test. The specificity was thus calculated to 100% (96.7 to 100%, 95% confidence interval).

### 3.5. Lysis Method

In these experiments, DNA from samples was first extracted and purified by using an automated device. For field use, a rapid and simple extraction method requiring few steps and materials may be needed. A quick sample lysis protocol was thus developed and verified on a subset of positive samples [skin lesions (n = 16) and throat swabs (n = 5) in transport medium], with Ct values ranging from 13.07 to 35.81 in qPCR.

The results indicated that the dry MPXV LAMP sensitivity was lower when the quick lysis method was used to prepare the samples before the analysis, in comparison with the LAMP assay performed with the reference method of DNA extraction (Table 1). With the reference method, 100% of the clinical samples with a Ct value in qPCR of up to 30.35 were detected as positive. For the lysis method, 100% detection was obtained for samples with Ct values in PCR of up to 26.40.

## 4. Discussion

The Mpox virus, currently of international concern, has been active in Africa for many decades. The 2023 outbreak on the continent, mediated by the new Ib variant and targeting mainly children, highlights once more the need for diagnostic tests able to detect the virus in human samples to limit human-to-human transmission and to help the management of infected persons more rapidly. The WHO has determined the laboratory-based NAAT (PCR) as the gold standard for the detection and confirmation of Mpox cases but also recommends the use of point-of-care (POC) assays to manage diagnosis more rapidly during outbreaks or in non-centralized laboratories [33]. Commercial molecular kits are already available for Mpox detection [34], such as the Cepheid Xpert^®^ Mpox, the Roche Molecular System cobas^®^ MPXV, and the Abbott Molecular Alinity m MPXV assay, which are listed on the WHO’s list of tests for emergency use. Of the 160 molecular tests already registered or commercially available, only 3 are true POC assays (Pluslife Monkeypox Virus Nucleic Acid Testing Card, PortNAT Monkeypox Virus Assay, and ProtonDx Dragonfly Skin Infection Viral Test Panel) [35,36,37]. In this context, Coris BioConcept is developing a rapid LAMP assay directed for use in the field as a POC test. To develop and verify this LAMP assay, Coris BioConcept worked in association with the Istituto Nazionale per le Malattie Infettive “Lazzaro Spallanzani”.

Two sets of primers were designed to target the ORF F3L and N4R regions of clades I and II (West African and Congo Basin clades). In silico analysis of clade Ia, Ib, IIa, and IIb genomes for the targeted F3L and N4R regions showed that only two point mutations were observed between clade I (a and b) and clade II (a and b) in the ORF F3L region. These point mutations are not located in the designed primers sequences. Although the positive samples processed during this study were all from clade IIb (2022 outbreak in Europe), it was expected that clades Ia, Ib, and IIa would be detected in addition to clade IIb with the same sets of LAMP primers.

Two versions of the assay in liquid format (frozen reagents), one targeting the ORF F3L and the other targeting the ORF F3L and the N4R region, were first compared for their sensitivity on clinical samples, corresponding to a range of Ct values in PCR from 12.85 to 35.91. It was observed that the version targeting two genes allowed for faster detection (average 1.3 min) with higher sensitivity (81.7% vs. 88.3%). The presence of primer dimers in the lowest concentrated samples led to the setting of a time-to-result cutoff value of 20 min.

To find the specifications for a POC test, stabilized reagents are needed to avoid the need for cold chain shipping and storage conditions. A protocol to dry a LAMP assay was developed and applied to the drying of the LAMP MPXV assay targeting the ORF F3L and N4R regions. The sensitivity of the dry assay, analyzed with the same samples as for the liquid version, was determined to be as good as for the liquid version of the same assay (88.3%). Because the samples were selected to represent a range of virus concentrations, as determined by qPCR, the sensitivity of the LAMP MPXV assay determined in this experiment represents a comparison with the PCR but not the clinical sensitivity of the assay. Considering that high virus titers could be expected during an outbreak, the clinical sensitivity should be expected to be higher. Concerning the specificity, none of the 112 tested samples that were positive for HPV DNA were detected. Sensitivity and specificity will be confirmed in the field in prospective studies in the future.

The efficiency of the dry test after long-term storage will be assessed by real-time stability studies (in progress). The compatibility of this dry LAMP MPXV assay with isothermal devices measuring fluorescence that are simpler and far cheaper than a thermocycler was already demonstrated. While fluorescence-based detection was used in this study, the use of conventional real-time thermocycler is not essential. As already demonstrated by Coris BioConcept, the dry LAMP MPXV assay is fully compatible with portable isothermal fluorometric devices, which are significantly more affordable. This feature makes the assay particularly suitable for point-of-care implementation in decentralized settings, enhancing its practical utility in low-resource contexts.

A simple and rapid lysis method was compared to a reference protocol involving an automated instrument. The simple and quick lysis method allowed for detection of the virus from all samples, with a Ct value in qPCR of under 26.4 and with a time-to-result only slightly higher than for the reference extraction method in most cases. This shows that the release of DNA is efficient. Unfortunately, although the simplified method allows for lysis of the sample and release of the virus DNA, the least concentrated samples (with the highest Ct value in qPCR) were either not detected or detected with a much higher time-to-result. Based on these observations, the strategy for the pre-analytic treatment of the samples was revised and a new lysis/extraction method that is simpler and faster is currently being developed.

A recent review article listed molecular diagnostic applications in development for Mpox diagnosis using qPCR (4 tests), RPA/RAA (Recombinase Polymerase Amplification/Recombinase-Aided Amplification—14 tests, including RPA-CRISPR-Cas12 assays), and LAMP (6 tests) [38]. One clear advantage of our dry LAMPOX assay is the fast time-to-result, with most of the samples (90.6%) being detected in less than 7 min. Indeed, most of the applications have a readout time above 30 min (all the LAMP assays described in the article), with the longest time-to-result being obtained with the qPCR method. One of these qPCRs targeting the F3L gene also targeted in the dry LAMPOX assay has a readout of more than 90 min and requires the use of a complex thermocycler instrument [27]. Only three RPA/RAA applications have detections as rapid as that of the dry MPXV LAMPOX assay. Amongst these fast RPA/RAA assays, one also targets F3L gene. This assay presented a sensitivity and a specificity of 100% on clinical samples but was composed of only three positive and seven negative ones [39]. The two other fast RPA/RAA assays target other genes and present high clinical sensitivity and specificity, but on a limited number of samples [40,41].

Concerning the LAMP assays described in the review, none are as fast as the dry LAMPOX assay (30 to 60 min for readout time). One of them targets the F3L gene associated with the A27L gene (visual turbidity detection) [42], and one another targets the N4R gene (colorimetric or fluorometric detections) [43]. These two LAMP assays were tested on simulated clinical samples for feasibility or on a small panel of clinical specimens (15), making it difficult to compare them with our results. No data are available about long-term stability of the reagents used in these applications. None of these applications are commercially available yet.

In conclusion, the dry MPXV LAMPOX assay’s high sensitivity and specificity combined with its ease of use, rapid time-to-result, and compatibility with fluorometric portable isothermal devices meet the needs for a POC test dedicated to detecting MPXV DNA in human samples. To complete the assay, a quick and simple preanalytical DNA extraction method (in its final stages of development) will soon be available.

## Figures and Tables

**Table 1:** Comparison of time-to-result obtained for DNA samples extracted with the reference method and samples treated with the quick lysis method. A time-to-result of above 30 min (maximal duration of the assay) means that no amplification was observed for this sample.

Sample Type	Cts in PCR	Time-to-Result in LAMP (min)	
		Reference Method	Lysis Method
Skin lesions	13.07	2.34	4.52
Skin lesions	13.80	4.33	3.46
Skin lesions	16.83	2.46	4.17
Skin lesions	18.54	2.83	1.58
Skin lesions	19.19	3.11	4.74
Throat swab	19.75	3.38	4.97
Throat swab	20.49	3.43	5.15
Skin lesions	20.49	2.70	5.87
Skin lesions	20.70	3.33	5.37
Skin lesions	22.61	3.83	5.64
Skin lesions	23.09	3.74	22.19
Skin lesions	24.06	3.76	5.62
Throat swab	26.40	4.46	6.46
Skin lesions	26.79	4.04	>30
Throat swab	28.68	4.67	>30
Skin lesions	28.95	4.58	>30
Skin lesions	29.96	4.54	19.94
Throat swab	30.33	5.55	>30
Skin lesions	30.35	4.83	22.99
Skin lesions	33.87	>30	>30
Skin lesions	35.81	28.99	>30

## Data Availability

The data presented in this study are available on request from the corresponding author.
